# Antimicrobial, Cytotoxicity and Phytochemical Screening of Jordanian Plants Used in Traditional Medicine

**DOI:** 10.3390/molecules15031811

**Published:** 2010-03-12

**Authors:** Wamidh H. Talib, Adel M. Mahasneh

**Affiliations:** Department of Biological Sciences, Faculty of Science, University of Jordan, Amman-11942, Jordan; E-Mail: altaei_wamidh@yahoo.com (W.H.T.)

**Keywords:** antimicrobial activity, cytotoxicity, plant extracts, MTT and TLC analysis

## Abstract

Antimicrobial activity and cytotoxicity of fifty one extracts of different parts of 14 plants were studied. Ethanol, methanol, aqueous, butanol, and *n*-hexane extracts were tested against three Gram negative, two Gram positive bacteria, and two fungi. Cytotoxicity and phytochemical screening were determined using MTT and TLC assays, respectively. Of the fifty one extracts, twenty two showed activities against different microorganisms with MICs ranging from 62.5 to 1000 µg/mL. The highest activity (100% inhibition) was for a butanol extract of *Rosa damascena* receptacles against *Salmonella typhimurium* and *Bacillus cereus* (MIC of 62.5 and 250 µg/mL) respectively. Butanol extract of *Narcissus tazetta* aerial parts and aqueous extract of *Rosa damascena* receptacles were both active against *Candida albicans* (MIC of 125 µg/mL). Methicillin-resistant *Staphylococcus aureus* was inhibited by butanol, aqueous extracts of *Rosa damascena* receptacles and butanol extract of *Inula viscosa* flowers (MIC of 500, 500, and 250 µg/mL) respectively. *Rosa damascena* receptacles and *Verbascum sinaiticum* flowers ethanol extract showed lowest cytoxicity against Vero cell line (IC_50 _of 454.11and 367.11). Most toxic was the ethanol extract of *Ononis hirta* aerial parts (IC_50 _72.50 µg/mL). Flavonoids and terpenoids were present in all plants. *Ononis hirta* and *Narcissus tazetta* contained alkaloids. The results validate the use of these plants and report for the first time bioactivity of *Rosa damascena* receptacles and further justifies the use of such screening programs in the quest for new drugs.

## 1. Introduction

The discovery of antibiotics has decreased the spread and severity of a wide variety of diseases. However, and as a result of their uncontrolled use, the efficiency of many antibiotics is being threatened by the emergence of microbial resistance to existing chemotherapeutic agents [1]. Bacteria and fungi are evolving numerous mechanisms to evade antimicrobial agents and the resistance to old and new antibiotics is rising in medical practice [2]. Bacterial strains such as methicillin-resistant *Staphylococcus aureus* (MRSA), penicillin-resistant *Streptococcus pneumonia* (PRSP), and vancomycin-resistant enterococci (VRE), in addition to the development of multidrug-resistant (MDR) bacterial strains [3], are just few examples that have made the search for new and novel antimicrobial substances among the first priorities within the quest for such materials. In addition the lack of new antifungal agents and the long-term use of antifungal drugs in the treatment of chronic fungal infections has caused the emergence of amphotericin-B and azole resistant *Candida* species [4]. Not to mention the fact that the use of some antibiotics is associated with side effects, including allergy, immune- suppression, and hypersensitivity [5]. For all these reasons, there is a pressing need to identify new and novel antimicrobial agents that would help in alleviating the problems of emerging resistant bacterial and fungal pathogens. Plant derived natural products represent an attractive source of antimicrobial agents since they are natural and affordable, especially for rural societies in poor developing countries [6] and also used in different formulations by different cultures as part of their materia medica heritage [2]. However, due to toxicity of some of the medicinal plants, great care should be observed in their prescription. Also plant derived agents may have different mechanisms of action than conventional drugs, and this could be of clinical importance in health care improvement [7]. Plants are rich sources of many bioactive secondary metabolites that have the potential to treat different afflictions. Examples of these compounds include flavonoids, phenols, phenolic glycosides, unsaturated lactones, sulphur compounds, saponins, cyanogenic glycosides and glucosinolates [8]. 

Jordan is relatively small country but it is characterized by having a great variation in wild plant species. Out of the 2,500 Jordanian plant species recorded, more than 100 species are listed as endemic [9] and 285 species are classified as medicinal plants [10]. However, little is known about the possible medical applications of such plants [10,11,12]. Thus the objective of this study was to evaluate the potential antibacterial and antifungal activities of 51 extracts of different parts of 14 plants used in popular medicine to alleviate different diseases. The plants (Table 1) were selected based on the information gathered from traditional healers, reputed informants and their inclusion in the Jordanian *materia medica* as sources of probable biological activity [13].

## 2. Results and Discussion

Jordanian plants have received more attention in the last few years since they are widely used in traditional medicine [14] and are characterized by high diversity and endemism [13]. All tested plants in this study are traditionally used as medicinal plants in different localities of Jordan. Their medicinal uses are summarized in Table 1.

**Table 1:** Ethnobotanical data about the studied plants.

Plant scientific name, family, voucher numbers	Local name	Parts used	Traditional and/or medicinal use	Mode of use
*Populus alba *L. Salicaceae, MAHAS 2	Al-Hoor al-abyad	Flowers	Depurative, tooth decay, skin lesions and herpes.	Decoction, infusion/oral
*Teucrium polium* L. Labiatae, MAHAS 9	Jeada	Aerial parts	Anti-inflammatory, spasm, flatulence, diabetes, necrosis and kidney stones	Infusion
*Salvia pinardi* L. Labiatae, MAHAS 10	Miramia	Aerial parts	Sedative, for wound healing and herpes	Infusion
*Syringa vulgaris* L. Oleaceae, MAHAS 11	Lailac	Aerial parts, seeds	Antihelminthic, anti-febrifuge, dry skin, and in the treatment of malaria	Infusion, topical
*Phagnalon rupstre *L*.* Asteraceae, MAHAS 12	Kadha	Aerial parts	Any disease of unknown reason, inflammation, rheumatism, migraine, depression, scalp infection.	Decoction, infusion/oral
*Inula viscos*a (L.) Ait. Compositae, MAHAS 13	Taioon	Flowers	Anthelmintic, for lung cancer, muscle relaxant	Decoction
*Luffa cylindrica* L. Araceae, MAHAS 14	Louf	Aerial parts	Treatment of cancer, post-delivery pain, inflammation, infections	Oral/ infusion
*Pterocephalus pulverulentus *Boiss Dipsacaceae, MAHAS 15	Abu Moch	Aerial parts	Ulceration	Gargle
*Mirabilis jalapa* L. Nyctaginaceae, MAHAS 16	Chap-Zarief	Aerial parts, roots, stems	Antiseptic, antiviral, fungicide, antiabortive, useful in epilepsy and chronic bronchitis	Oral/infusion
*Narcissus tazetta* L. Amaryllidaceae, MAHAS 17	Narjes	Aerial parts,flowers	Anticancer, antiinflammatory, memorigenic, and sedative	Infusion
*Verbascum sinaiticum* L. Scrophulariaceae MAHAS 18	Al-Omaya	Flowers, aerial parts	Neural pain, herpes and bronchitis.	Decoction
*Rosa damascena* Mill. Rosaceae, MAHAS 19	Ward Demashqi	Receptacles, seeds	Antibacterial, treatment of cardiac diseases, colon cancer	Decoction
*Ononis sicula* Desf. Fabaceae, A O 298	Shibreq	Aerial parts	Skin cancer, lesions	Topical/wash
*Ononis hirta *L. Fabaceae, A O 297	Showk Al-Jamal	Aerial parts	Skin cancer, necrosis, herpes, cold sores	Mouth and skin wash

In this study we evaluated the antimicrobial activity of fifty one extracts from fourteen plants used by traditional healers to treat different ailments. MIC (minimum inhibitory concentration), MBC (minimum bactericidal concentration), cytotoxicity and TLC were determined for extracts that showed promising activity against tested microorganisms.

The results of the antimicrobial testing of 1 mg/mL ethanol extracts of the tested plants revealed that eight plants have potential antimicrobial activity (> 50% inhibition using the microplate assay) against one or more of the tested microorganisms (Table 2) where significant activities are typed in boldface. These plants are *Ononis hirta* (aerial parts), *Verbascum sinaiticum* (flowers), *Inula viscosa* (flowers), *Ononis sicula* (aerial parts), *Narcissus tazetta* (aerial parts), *Rosa damascena* (receptacles), *Phagnalon rupustre* (aerial parts), and *Teucrium polium* (aerial parts). Al-Fatimi, *et al*. [15] in similar study, screened 90 plant crude extracts and found 67 of these to be active against both the Gram positive and Gram negative bacteria tested.

The ethanol extract of the eight active plants showed different activities against an array of Gram positive, Gram negative bacteria and fungi. All of these plant extracts were active against *Salmonella typhimurium*, while no activity was detected against *Aspergillus niger*, a filamentous fungus, but some extracts were active against the yeast *Candida albicans*. *Escherichia coli* was not affected by any of the ethanol extracts except for that of *Rosa damascena* receptacles. This extract and the *Narcissus tazetta* (aerial parts) extract were the only ethanol extracts that showed any substantial antifungal activity against C*andida albicans*, which is very interesting and is reported here for the first time. Preliminary screening of some Colombian medicinal plants showed *in vitro* antimicrobial activity of several crude extracts of such plants [16]. Methicillin resistant *Staphylococcus aureus* (MRSA) was susceptible to the ethanol extracts of *Rosa damascena* receptacles (95% inhibition), *Inula viscosa* flowers (92% inhibition, and *Verbascum sinaiticum *flowers (70% inhibition).

**Table 2:** Antimicrobial activity (% Inhibition) of 1mg/mL ethanolic plant extracts.

Plant	% Inhibition						
	*P.a*	*E.c*	*S.t*	*B.c*	*MRSA*	*C.a*	*A.n*
***Inula viscosa*** **(f)**	**96.12**	9.88	**99.12**	**101.57**	**92.43**	22.13	17.46
*Rosa damascena* (s)	25.50	18.50	11.45	17.97	45.95	31.30	25.67
***Rosa damascena*** **(r)**	**80.49**	**60.69**	**100.82**	**101.09**	**95.75**	**98.87**	30.72
***Ononis hirta*** **(a.p)**	45.89	12.49	**93.40**	81.91	-72.82	40.23	17.34
***Ononis sicula*** **(a.p)**	**60.75**	21.28	**79.03**	**74.67**	-60.23	25.40	19.68
*Syringa vulgaris* (s)	43.56	-1.41	-33.60	-20.75	-12.76	12.92	14.18
*Syringa vulgaris* (a.p)	46.98	-8.60	-2.22	-34.08	-42.32	16.74	23.25
*Populus alba* (f)	40.12	31.38	39.11	43.00	34.65	28.27	18.25
***Teucrium polium*** **(a.p)**	**56.54**	25.84	**80.08**	**94.27**	3.53	19.33	16.45
*Salvia pinardi * (a.p)	28.71	-68.30	43.99	45.38	28.73	-49.76	-50.68
***Phagnalon rupstre*** **(a.p)**	19.51	-24.10	**77.86**	**71.71**	-63.28	-5.66	-8.70
*Luffa cylindrica* (a.p)	9.09	-35.07	43.93	39.20	38.38	5.34	0.87
***Verbascum sinaiticum*(f)**	**58.31**	18.02	**99.42**	-56.69	**70.54**	35.46	29.57
*Narcissus tazetta* (f)	46.98	21.93	-32.65	-36.61	-30.08	12.34	1.60
***Narcissus tazetta*** **(a.p)**	**52.23**	44.08	**70.50**	**75.93**	48.96	**99.23**	5.69
*Mirabilis jalapa *(ro)	35.48	26.93	-14.02	-11.46	13.17	27.97	10.11
*Mirabilis jalapa* (a.p)	13.75	5.32	-53.86	-56.70	-33.92	17.90	2.54
*Mirabilis jalapa* (st)	40.56	17.37	-28.27	15.26	-8.51	16.87	-5.60
*Pterocephalus pulverulentus* (a.p)	43.02	5.65	3.45	40.82	26.24	28.98	13.32

Microbial species: Methicillin resistant *S*. *aureus* (MRSA); *Bacillu cereus* (B.c); *Escherichia coli* (E.c); *Pseudomonas aeruginosa* (P.a); *Salmonella typhimurium* (S.t) C*andida albicans* (C.a); *Aspergillus niger* (A.n). Plants in bold which showed inhibition of some microorganisms ranging from 50–100 % were selected for MIC determination. f: flowers, a.p: aerial parts, ro: root, r: receptacles, st: stem, s: seeds. Penicillin, tetracycline, and nystatin were used as positive controls. DMSO was also included being the solvent for the extracts.

All the ethanol extracts were active against *Pseudomonas aeruginosa*, except O*nonis hirta* (aerial parts) and *Phagnalon rupstre* (aerial parts). On the other hand, the ethanol extract of *Verbascum sinaiticum* (flowers) was the only ethanol extract that showed no activity against *Bacillus cereus*. In the second stage of screening, MIC and MBC values of ethanol, butanol, methanol, aqueous, and *n*-hexane extracts of the active plant extracts were determined (Table 3). 

The most potent extract was the butanol one of *Rosa damascena* receptacles, which had MIC values of 62.5, 125, 250, 500, and 1000 against *Salmonella typhimurium*, *Pseudomonas aeruginosa*, *Bacillus cereus*, and C*andida albicans*, respectively.

Other plants showed different MIC values ranging from 125 to > 1,000 µg/mL against different microorganisms, with the activity being most prevalent in the butanol and aqueous extracts, except for O*nonis hirta* aerial parts, and *Teucrium polium* aerial parts whose *n*-hexane extract showed interesting activity (MIC of 125µg/mL) against *Pseudomonas aeruginosa*.

**Table 3:** Minimum inhibitory concentration (MIC) in µg/mL of plant extracts.

Plant Extract		Test Microorganisms					
		MRSA	E.c	P.a	S.t	B.c	C.a
*R.d* (r)	Eth.	> 1000	> 1000	> 1000	> 1000	1000	250
	But.	500	> 1000	125	62.5	250	1000
	Aqu.	500	> 1000	250	250	500	125
	Met.	> 1000	> 1000	> 1000	> 1000	> 1000	1000
	Hex.	> 1000	> 1000	> 1000	> 1000	> 1000	> 1000
*I.v* (f)	Eth.	> 1000	> 1000	> 1000	> 1000	1000	ND
	But.	250	> 1000	500	125	250	ND
	Aqu.	> 1000	> 1000	250	> 1000	> 1000	ND
	Met.	500	> 1000	1000	> 1000	1000	ND
	Hex.	1000	> 1000	> 1000	1000	500	ND
*P.r* (ap)	Eth.	> 1000	> 1000	> 1000	> 1000	> 1000	ND
	But.	> 1000	> 1000	> 1000	1000	> 1000	ND
	Aqu.	> 1000	> 1000	> 1000	> 1000	> 1000	ND
	Met.	> 1000	> 1000	> 1000	> 1000	> 1000	ND
	Hex.	> 1000	> 1000	> 1000	> 1000	> 1000	ND
*O.h* (ap)	Eth.	> 1000	> 1000	> 1000	> 1000	> 1000	ND
	But.	> 1000	> 1000	500	> 1000	> 1000	ND
	Aqu.	> 1000	> 1000	125	> 1000	> 1000	ND
	Met.	> 1000	> 1000	> 1000	> 1000	> 1000	ND
	Hex.	> 1000	> 1000	125	> 1000	> 1000	ND
*T.p *(ap)	Eth.	> 1000	> 1000	> 1000	> 1000	> 1000	ND
	But.	> 1000	> 1000	250	> 1000	> 1000	ND
	Aqu.	> 1000	> 1000	> 1000	> 1000	> 1000	ND
	Met.	> 1000	> 1000	1000	> 1000	> 1000	ND
	Hex.	> 1000	> 1000	125	> 1000	> 1000	ND
*O.s *(ap)	Eth.	> 1000	> 1000	> 1000	> 1000	> 1000	ND
	But.	> 1000	> 1000	500	> 1000	> 1000	ND
	Aqu.	> 1000	> 1000	> 1000	> 1000	> 1000	ND
	Met.	> 1000	> 1000	> 1000	> 1000	> 1000	ND
	Hex.	> 1000	> 1000	> 1000	> 1000	> 1000	ND
*V.s *(f)	Eth.	> 1000	> 1000	> 1000	1000	1000	ND
	But.	> 1000	> 1000	> 1000	1000	1000	ND
	Aqu.	1000	1000	> 1000	1000	1000	ND
	Met.	> 1000	> 1000	> 1000	1000	1000	ND
	Hex.	> 1000	> 1000	> 1000	> 1000	> 1000	ND
*N.t *(a.p)	Eth.	> 1000	> 1000	> 1000	> 1000	> 1000	500
	But.	> 1000	> 1000	> 1000	> 1000	> 1000	125
	Aqu.	> 1000	> 1000	> 1000	> 1000	> 1000	500
	Met.	> 1000	> 1000	> 1000	> 1000	> 1000	> 1000
	Hex.	> 1000	> 1000	> 1000	> 1000	> 1000	> 1000
Tetracycline		ND	8	8	4	ND	ND
Penicillin G		10	ND	ND	ND	8	ND
Nystatin		ND	ND	ND	ND	ND	8

Plants: *R.d* (r): *Rosa damascena* (receptacles), *I.v* (f): *Inula viscosa* (flowers), *P.r *(ap): *Phagnalon rupstre* (aerial parts), *O.h* (ap): *Ononis hirta* (aerial parts), *T.p *(ap): *Teucrium polium* (aerial parts), *O.s *(ap): *Ononis sicula* (aerial parts), *V.s *(f): *Verbascum sinaiticum*(flowers), *N.t *(a.p): *Narcissus tazetta* (aerial parts). Microbial species: Methicillin resistant *Staphylococcus aureus* (MRSA); *Bacillus cereus* (B.c); *Escherichia coli* (E.c); *Pseudomonas aeruginosa* (P.a); *Salmonella typhimurium* (S.t) C*andida albicans* (C.a). ND: not determined. Values for *Aspergillus niger* were all above 1,000 µg/mL hence we did not include them. Pure tetracycline, penicillin, and nystatin were included for comparative purposes.

Successive isolation of active compounds from plants depends upon the type of solvent used in extraction procedure [2]. Our results showed that the activity is mainly concentrated in the butanol and aqueous extracts, indicating that the potential antimicrobial compounds were in the high polarity fractions. Such results are in agreement with other data reported in literature. Mahasneh and EL-Oqlah [11] concluded that butanols extracts have superior antimicrobial activity compared with other ones. Buwa and Staden [17] reported that the aqueous extracts were more active against bacteria compared with ethanol and ethyl acetate extracts. 

Water is usually the main solvent used by traditional healers to prepare plant extracts. However, we found in this study that plant extracts derived from ethanol produced more consistent antimicrobial activity and this conclusion is shared also by other researchers [18].

It is generally expected that more plant extracts would be active against Gram positive bacteria compared with Gram negative bacteria [19]. However, in the present study, all eight active plants showed activity against both Gram positive and Gram negative bacteria (Table 2) and it is worthy to report that among the Gram positive bacteria tested are methicillin-resistant *Staphylococcus aureus* and *Bacillus cereus*, which are frequently implicated in food poisoning episodes. Such activity of plant extracts may be a result of the presence of broad spectrum antibacterial compounds or general metabolic toxins [20]. Both possibilities need further investigation using purified extracts in order to identify the specific active compounds which would be followed up later in our laboratory.

Most clinical studies showed that the mortality from opportunistic fungal diseases is above 50% and may reach 95% in bone marrow transplant recipients [21]. Due to the toxicity and limited spectrum of the commonly used antifungal drugs [22] there is a need for the isolation of new antifungal agents especially from plant extracts. In this context, the searching of plant extracts for antifungal agents has been a valid strategy [23,24] and our antifungal tests represent a modest contribution in this regard.

Extracts of *Rosa damascena* receptacles and *Narcissus tazetta* aerial parts showed a potential antifungal activity against C*andida albicans* (Table 3). The activity was rather prominent in the aqueous and ethanol extracts of *Rosa damascena* receptacles with MIC values of 125 and 250 µg/mL, respectively. *Narcissus tazetta* aerial parts, meanwhile, showed activity in butanol, aqueous, and ethanol extract form with MIC values of 125, 500, and 500 µg/mL, respectively. These values, if compared with pure nystatin with a recorded MIC value of 8 µg/mL, appear to be higher but this is expected where our test used crude extracts. 

The MBC and MFC (minimum fungicidal concentration) of all active plant extracts showed values higher than 1000 µg/mL (Data not shown), indicating a bacteriostatic and fungistatic behavior of these extracts [21].

The results of this study showed high antimicrobial activity of *Rosa damascena* receptacles extracts and in previous studies the essential oils of the flower of this plant exhibited antibacterial activities against *Staphylococcus aureus* and *Propionibacterium acnes* [25,26]. Our study is thus the first to evaluate the antimicrobial activity of rose receptacles compared with the previous studies that focused on the essential oils extracted from rose petals. 

The cytotoxicity of the active plant extracts was examined against Vero cell line using the MTT assay (Table 4). The lowest toxicity was recorded for *Rosa damascena* receptacles with an IC_50_ value of 454.11 µg/mL, while the highest toxicity was that of *Ononis hirta* aerial parts with an IC_50_ value of 72.50 µg/mL. Toxicity data reported in this study could potentially be used to assess the anticancer activities of such plant extracts [27] which is not in the scope of this paper but being followed up in another study in our laboratory.

**Table 4:** Cytotoxicity of the ethanolic extract of plants with antimicrobial potential.

Plant	IC_50_ (μg/mL) ± S.D
*Inula viscosa* (flowers)	202.43 ± 3.70
*Rosa damascena* (receptacles)	454.11± 2.87
*Ononis hirta* (aerial parts)	72.50 ± 1.34
*Ononis sicula* (aerial parts)	325.17 ± 3.00
*Phagnalon rupstre* (aerial parts)	197.23 ± 4.2
*Narcissus tazetta* (aerial parts)	131.01 ± 5.20
*Verbascum sinaiticum*(flower)	367.11 ± 2.70
*Teucrium polium* (aerial parts)	110.03 ± 1.50

Phytochemical screening of active plant extracts (Table 5) showed the presence of flavonoids and terpenoids in all tested plants, while alkaloids were detected in *Ononis hirta* aerial parts and *Narcissus tazetta *aerial parts. The *in vitro* antimicrobial activity of several phytochemicals including flavonoids, tannins, coumarins, terpenoids and alkaloids has been reported [28]. However, in order to consider these phytochemicals as really promising antimicrobial agents different studies including cytotoxicity determination, mechanism of action, interaction with other compounds, and pharmacological applicability should be performed [29]. 

Flavonoids are present widely in the plant kingdom and posses many functions including anti-inflammatory, antimicrobial, enzyme inhibition, antioxidant and antitumor [30]. Previous studies reported the antimicrobial activity of many flavonoids rich plants that have broad antimicrobial activity [31]. And some plants that have flavonoids active against *Candida albicans* were also reported [32,33]. Additionally flavonoids are characterized by low toxicity since they are widely distributed in edible plants [30]. The results of this part of the preliminary phytochemical screening is in agreement with these findings because flavonoids were detected in all active plant extracts where some showed low toxicity toward Vero cell line.

**Table 5:** Thin layer chromatography analysis of most active plants extracts

Plant	alkaloids	flavonoids	terpenoids
*Ononis hirta* (aerial parts)	+	+	+
*Verbascum sinaiticum* (flowers)	-	+	+
*Inula viscosa* (flowers)	-	+	+
*Ononis sicula* (aerial parts)	-	+	+
*Narcissus tazetta* (aerial parts)	+	+	+
*Rosa damascena* (receptacles)	-	+	+
*Phagnalon rupustre* (aerial parts)	-	+	+
*Teucrium polium* (aerial parts)	-	+	+

These results, showing high antimicrobial potential of some plant extracts against different bacteria and fungi combined with cytotoxicity testing against a Vero cell line, especially these of *Rosa damascena* receptacles, open the door for potential clinical application which merit further analysis and investigation.

## 3. Conclusions

Continuous efforts in search of new and novel bioactive materials encouraged us to study the activity of the aforementioned plant extracts toward an array of microorganisms. Results showed for the first time the potential activity of *Rosa damascena* receptacles among other plant extracts against Gram positive and negative bacteria and fungi. Cytotoxicity of these extracts toward Vero cell line also necessitates further investigations. However, the results validate the use of some of these plants in folk medicine.

## 4. Experimental

### 4.1. Plant material

Plants were collected from the Amman and Ajloun areas in Jordan. The taxonomic identity of each plant was authenticated by Prof. Ahmad EL-Oqlah (Department of Biological Sciences, Yarmouk University, Irbid, Jordan) and Prof. Dawud EL-Eisawi (Department of Biological Sciences, University of Jordan, Amman, Jordan). Voucher specimens were deposited in the Department of Biological Sciences, University of Jordan, Amman, Jordan (Table 1).

### 4.2. Plant extraction and fractionation

Different plant samples were dried at room temperature and were finely ground. Suitable amounts of the powdered plant materials were soaked in 95% ethanol (1 L per 100 g) for two weeks. The crude ethanolic extracts were obtained after the solvent was evaporated at 40 °C to dryness under reduced pressure using a rotary evaporator (Buchi R-215, Switzerland). The residues were further subjected to solvent-solvent partitioning between chloroform and water. The dried chloroform extract was also partitioned between *n*-hexane and 10% aqueous methanol, while the butanol extract was fractionated from the aqueous extract [11]. All solvents were evaporated to dryness under reduced pressure to produce the crude extracts which were collected and stored at -20 °C for further testing. 

### 4.3. Microbial strains

The bacterial strains used in this study were *Escherichia coli* (ATCC 25922), *Salmonella typhimurium* (ATCC14028), *Pseudomonas aeruginosa* (Clinical isolate), Methicillin resistant *Staphylococcus aureus* (MRSA Clinical isolate), *Bacillus cereus* (Toxigenic strain TS), *Candida albicans* (ATCC 90028), and *Aspergillus niger* (ATCC 16404).

### 4.4. Antibacterial assay.

The micro-titer plate dilution method [7] was used to determine MIC (minimum inhibitory concentration) and MBC (minimum bactericidal concentration) values for the plants under study. Sterile 96-well microplates (Nunc, Denmark) were used for the assay. Stock plant extracts were dissolved in DMSO so that the final concentrations in micro wells were less than 1% DMSO and solvent controls were run at these concentrations. Positive controls of penicillin and tetracycline (Oxoid, UK) were prepared at the following concentrations 0.5, 1, 2, 4, 8, 16, 32, 64, and 128 μg/mL. 1% DMSO was used as a solvent control. Plant extracts were diluted to twice the desired initial test concentration (1 mg) with Muller Hinton broth (MHB) (Oxoid, UK). All wells, except the first were filled with MHB (50 μL). Plant extract (100 μL) was added to the first well and serial two-fold dilutions were made down to the desired minimum concentration (32 μg). An over-night culture of bacteria suspended in MHB was adjusted to turbidity equal to 0.5 McFarland standard. The plates were inoculated with bacterial suspension (50 μL/well) and incubated at 37 °C for 24 h. Then the turbidity was measured using micro-plate reader (Tecan, Austria) at 620 nm wavelength. The assay was conducted in two stages. In the first stage the antimicrobial activity of 1mg/mL of the ethanol extract of each plant was tested against all test microorganisms. Percentage inhibition was calculated using the following formula:


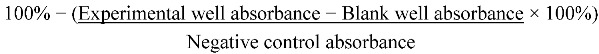


Plant extracts that showed more than 50% inhibition were selected for further testing in stage 2. In the second stage, MIC and MBC were determined for ethanol, aqueous, butanol, methanol, and *n*-hexane extracts of each active plant. MIC was determined as the lowest concentration of plant extract that inhibit the growth of each microorganism. MBC was determined as the lowest concentration of plant extract that prevent the growth of bacteria after sub-culturing on MH agar plates.

### 4.5. Antifungal assay

The same procedure was used to determine the antifungal activity of plant extracts against *Candida albicans*. The medium used was RPMI-1640 [34] supplemented with L-glutamine and 2% glucose buffered to pH 7 with 0.165 mol/L morpholinepropanesulfonic acid (MOPS). The yeast cells were suspended in 0.85% saline, with an optical density equivalent to 0.5 McFarland standard, and diluted 1:100 in RPMI-1640 to obtain the working concentration [34]. After 24 h incubation with plant extracts, the turbidity was measured at 530 nm wavelength using a spectrophotometer (Das, Italy).

The conidial viability assay was used to determine the antifungal activity of plant extracts against *Aspergillus niger* [35]. Briefly, RPMI-1640 containing 0.025% Tween 20 was used to wash *Aspergillus* colonies. The resulted suspension was adjusted to 80–82% transmission using spectrophotometer at 530 nm wave length. The adjusted suspension was diluted 1:50 using RPMI-1640 and used to inoculate the micro-plate containing serial twofold dilution of the plant extracts. The micro-plates were incubated with plant extracts for 48 h at 35 °C then the turbidity was measured at 530 nm wavelength. Nystatin (Oxoid, UK) with the following concentrations 0.5, 1, 2, 4, 8, 16, 32, 64, and 128 μg/mL was used as a positive control and 1% DMSO was used as a solvent control.

### 4.6. Cytotoxicity study

The Vero cell line (kindly provided by Dr. Mona Hassuneh; Department of Biological Sciences, University of Jordan, Amman, Jordan) was used to determine the cytotoxicity of the plants that showed a promising antimicrobial activity. Vero cells were grown in Minimum Essential Medium Eagle (MEM) (Gibco, UK) supplemented with 10% heat inactivated fetal bovine serum (Gibco, UK), 29 μg/mL L-glutamine, and 40 µg/mL gentamicin and were incubated in a humidified atmosphere of 5% CO_2_ at 37 °C. The cytotoxicity of plant extracts was measured using MTT (3-(4,5- dimethylthiazol-2-yl)-2,5-diphenyltetrazolium bromide) assay (Promega, USA). The assay detects the reduction of MTT by mitochondrial dehydrogenase to a blue formazan product, which reflects the normal function of mitochondria and cell viability [36]. Eleven concentrations (500, 400, 300, 200, 100, 150, 100, 50, 25, 15, 5 μg/mL) were prepared from each ethanolic extract of plants that showed antimicrobial activity. Exponentially growing Vero cells were washed and seeded at 17,000 cells/well (in 200 μL of growth medium) in 96 well microplates (Nunc, Denmark). After 24 h incubation, a partial monolayer was formed then the media was removed and 200 μL of the medium containing the plant extract (initially dissolved in DMSO) were added and re-incubated for 48 h. Then 100 μL of the medium were aspirated and 15 μL of the MTT solution were added to the remaining medium (100 μL) in each well. After 4 h contact with the MTT solution, blue crystals were formed. 100 μL of the stop solution were added and incubated further for 1h. Reduced MTT was assayed at 550 nm using a microplate reader (Das, Italy). Culture medium containing 0.1% DMSO was used as a solvent control. Untreated cells were used as a negative control. 

### 4.7. Phytochemical screening

Qualitative thin layer chromatography (TLC) was conducted for extracts that showed potential antimicrobial activity against the tested microorganisms. Aliquots (50–75 μL) of the ethanolic extract were applied 1 cm from the base of the TLC plates (0.25 mm, Nacherey-Nagel, Germany). Serial gradients of chloroform and methanol (from 0 % to 100 %) were used as eluents. Development of the chromatograms was done in a closed tank in which the atmosphere had been saturated with the eluent vapor by lining the tank with filter paper wetted with the eluent. For flavonoids and terpenoids identification, plates were sprayed with *p*-anisaldehyde/sulfuric acid reagent and carefully heated at 105^o^C for optimal color development [23]. For alkaloids detection, plates were sprayed with iodoplatinic acid and dried in the fume hood.

## References

[1] Cowan M.M. (1999). Plant products as antimicrobial agents. Clin. Microbiol. Rev..

[2] Pareke J., Chanda S. (2007). *In vitro* screening of antibacterial activity of aqueous and alcoholic extracts of various Indian plant species against selected pathogens from Enterobacteriaceae. AJMR.

[3] Alanis A.L. (2005). Resistance to antibiotics: are we in the post-antibiotic era?. Arch. Med. Res..

[4] Ficker C.E., Arnason J.T., Vindas P.S., Alvarez L.P., Akpagana K., Gbeassor M. (2005). Inhibition of human pathogenic fungi by ethnobotanically selescted plant extracts. Mycoses.

[5] Ahmed I., Mehmood Z., Mohammad F. (1998). Screening of some Indian medicinal plants for their antimicrobial properties. J. Ethnopharmacol..

[6] Ghosh A., Das B.K., Roy A., Mandal B., Chandra G. (2008). Antibacterial activity of some medicinal plant extracts. J. Nat. Med..

[7] Eloff J.N. (1998). Which extractant should be used for the screening and isolation of antimicrobial components from plants?. J. Ethnopharmacol..

[8] Quiroga E.N., Sampietro A.R., Vattuone M.A. (2001). Screening antifungal activities of selected medicinal plants. J. Ethnopharmacol..

[9] Al-Eisawi D. (1982). List of Jordan vascular plants. Mitteilungen Botanik Munchen..

[10] Aburjai T., Hudaib M., Tayyem R., Yousef M., Qishawi M. (2007). Ethnopharmacological survey of medicinal herbs in Jordan, the Ajloun heights region. J. Ethnopharmacol..

[11] Mahasneh A., El-Oqlah A. (1999). Antimicrobial activity of extracts of herbal plants used in the traditional medicine in Jordan. J. Ethnopharmacol..

[12] AL-Hussaini R., Mahasneh A.M. (2009). Microbial growth and quorum sensing antagonist activities of herbal plant extracts. Molecules.

[13] Al-Qura’n S. (2009). Ethnopharmacological survey of wild medicinal plants in Showbak, Jordan. J. Ethnopharmacol..

[14] Hudaib M., Mohammad M., Bustanji Y., Tayyem R., Yousef M., Abuirjeie M., Aburjai T. (2008). Ethnopharmacological survey of medicinal plants in Jordan, Mujib nature reserve and surrounding area. J. Ethnopharmacol..

[15] Al-Fatimi M., Wurster M., Shröder G., Lindequist U. (2007). Antioxidant, antimicrobial, and cytotoxic activities of selected medicinal plants from Yeme. J. Ethnopharmacol..

[16] Rojas J.J., Ochoa V.J., Ocampo S.A., Munoz J.F. (2006). Screening for antimicrobial activity of ten medicinal plants used in Colombian folkloric medicine. A possible alternative in the treatment of non-nosocomial infections. BMC Complement. Altern. Med..

[17] Buwa L.V., Staden J.V. (2006). Antibacterial and antifungal activity of traditionalmedicinal plants used against venereal diseases in South Africa. J. Ethnopharmacol..

[18] Aliero A.A., Afolayan A.J. (2006). Antimicrobial activity of *Solanum tomentosum*. Afr. J. Biotechnol..

[19] Mc Cutchon A.R., Ellis S.M., Hancock R.E.W., Towers G.H.N. (1992). Antibiot screening of medicinal plants of the British Columbian native peoples. J. Ethnopharmacol..

[20] Srinivasan D., Nathan S., Suresh T., Perumalsamy L.P. (2001). Antimicrobial activity of certain Indian medicinal plants used in folkloric medicine. J. Ethnopharmacol..

[21] Regasini L.O., Cotinguiba F., Morandim A.A., Kato M.J., Scorzoni L., Mendes-Gianni M.J., Bolzani V.S., Furian M. (2009). Antimicrobial activity of *Piper arboretum* and *Piper tuberculatum* (Piperaceae) against opportunistic yeasts. Afr. J. Biotechnol..

[22] Helmerhorst E.J., Reijnders I.M., Hof W.V.T., Smith I.S., Veerman E.C.J., Amerongen A.V.N. (1999). Amphotericin B and fluconazole-resistant *Candida* spp, *Aspergillus fumigatus* and other newly emerging pathogenic fungi are susceptible to basic antifungal peptides. Antimicrib. Agents Chemother..

[23] Masoko P., Mmushi T.J., Mogashoa M.M., Mokgotho M.P., Mampuru L.J., Howard R.L. (2008). *In vitro* evaluation of the antifungal activity of *Sclerocarya birrea* extracts against pathogenic yeast. Afr. J. Biotechnol..

[24] Adegoke A.A., Adebayo-Tayo B.C. (2009). Antimicrobial activity and phytochemical analysis of leaf extracts of *Lasienthera africanum*. Afr. J. Biotechnol..

[25] Aridogan B.C., Baydar H., Kaya S., Demirci M., Ozbasar D., Mumca E. (2002). Antimicrobial activity and chemical composition of some essential oils. Arch. Pharm. Res..

[26] Tsai T., Tsai T.H., Wu W., Tseng J., Tsai P. (2010). *In vitro* antimicrobial and anti- inflammatory effects of herbs against *Proionibacterium acnes*. Food Chem..

[27] Kornienko A., Evidente A. (2008). Chemistry, biology, and medicinal potentials of Narciclasine and its congeners. Chem. Rev..

[28] Ncube N. S., Afolayan A.J., Okoh A.I. (2008). Assessment techniques of antimicrobial properties of natural compounds of plant origin: current methods and future trends. Afr. J. Biotechnol..

[29] Alviano D.S., Alviano C.S. (2009). Plant extracts: search for new alternatives to treat microbial diseases. Curr. Pharm. Biotechnol..

[30] Cushnie T.P., Lamb A.J. (2005). Antimicrobial activity of flavonoids. Int. J. Antimicro. Ag..

[31] Rauha J., Remes S., Heinonen M, Hopia A., Kahkonen M., Pihlaja K., Vuorela H., Vuorela P. (2000). Antimicrobial effects of Finnish plant extracts containing flavonoids and other phenolic compounds. Int. J. Food Microbiol..

[32] Valsaraj R., Pushpangadan P., Smitt U.W., Adsersen A., Christensen S.B., Sittie A., Nyman U., Nielsen C., Olsen C.E. (1997). New anti-HIV-1, antimalarial, and antifungal compounds from *Terminalia bellerica*. J. Nat. Prod..

[33] Wachter G.A., Hoffmann J.J., Furbacher T., Blake M.E., Timmmermann B.N. (1999). Antibacterial and antifungal flavanones from *Eysenhardtia texana*. Phytochemistry.

[34] Scorzoni L., Benaducci T., Almeida A.M.F., Silva D.H.S., Bolzani V.S., Mendes- Giannini M.J.S. (2007). Comparative study of disk diffusion and microdilution methods for evaluation of antifungal activity of natural compounds against medical yeast *Candida *spp and *Cryptococcus* spp. J. Basic. Appl. Pharm. Sci..

[35] Balajee S.A., Marr K.A. (2002). Conidial viability assay for rapid susceptibility testing of *Aspergillus* species. J. Clin. Microbiol..

[36] Lau C.S., Ho C.Y., Kim C.F., Leung K.N., Fung K.P., Tse T.F., Chan H.L., Chow M.S. (2004). Cytotoxic activities of *Coriolus versicolor* (Yunzhi) extract on human leukemia and lymphoma cells by induction of apoptosis. Life Sci..

